# The Australian Multiple Sclerosis (MS) Immunotherapy Study: A Prospective, Multicentre Study of Drug Utilisation Using the MSBase Platform

**DOI:** 10.1371/journal.pone.0059694

**Published:** 2013-03-19

**Authors:** Vilija G. Jokubaitis, Tim Spelman, Jeannette Lechner-Scott, Michael Barnett, Cameron Shaw, Steve Vucic, Danny Liew, Helmut Butzkueven, Mark Slee, on behalf of the Australian MSBase Study Group

**Affiliations:** 1 Melbourne Brain Centre (RMH), Department of Medicine, The University of Melbourne, Parkville, Victoria, Australia; 2 Department of Neurology, Royal Melbourne Hospital, Parkville, Victoria, Australia; 3 Department of Neurology, John Hunter Hospital, Newcastle, New South Wales, Australia; 4 Hunter Medical Research Institute, University of Newcastle, Newcastle New South Wales, Australia; 5 Brain Mind Research Institute, Sydney, New South Wales, Australia; 6 Department of Neuroscience, Geelong Hospital, Geelong, Victoria, Australia; 7 Department of Neurology, Westmead Hospital, Westmead, New South Wales, Australia; 8 Department of Neurology, Box Hill Hospital, Monash University, Box Hill, Victoria, Australia; 9 Flinders University and Medical Centre, Adelaide, South Australia, Australia; University Hospital La Paz, Spain

## Abstract

**Objective:**

To prospectively characterise treatment persistence and predictors of treatment discontinuation in an Australian relapsing-remitting multiple sclerosis (RRMS) population.

**Methods:**

Tertiary MS treatment centres participating in the MSBase registry prospectively assessed treatment utilisation, persistence, predictors of treatment discontinuation and switch rates. Multivariable survival analyses were used to compare treatment persistence between drugs and to identify predictors of treatment discontinuation.

**Results:**

1113 RRMS patients were studied. Patients persisted on their first disease-modifying therapy (DMT) for a median of 2.5 years. Treatment persistence on GA was shorter than on all IFNβ products (p<0.03). Younger age at treatment initiation and higher EDSS were predictive of DMT discontinuation. Patients persisted on subsequent DMTs, for 2.3 years. Patients receiving natalizumab (NAT) as a subsequent DMT persisted longer on treatment than those on IFNβ or GA (p<0.000). The primary reason for treatment discontinuation for any drug class was poor tolerability. Annualised switch or cessation rates were 9.5–12.5% for individual IFNβ products, 11.6% for GA and 4.4% for NAT.

**Conclusion:**

This multicentre MS cohort study is the first to directly compare treatment persistence on IFNβ and GA to NAT. We report that treatment persistence in our Australian RRMS population is short, although patients receiving IFNβ as a first DMT persisted longer on treatment than those on GA. Additionally, patients receiving NAT as a subsequent DMT were more likely to persist on treatment than those switched to IFNβ or GA. EDSS and age at DMT initiation were predictive of DMT discontinuation. Treatment intolerance was the principal reason for treatment cessation.

## Introduction

Multiple Sclerosis (MS) is an inflammatory and degenerative disease of the central nervous system. For most patients with MS, the initial disease course features relapses and remissions (RRMS), whereas the later disease course is characterised by the progressive accumulation of disability. Early in the course of RRMS, parenteral disease modifying therapies (DMT), such as interferon-beta (IFNβ), glatiramer acetate (GA) or natalizumab (NAT) reduce the relapse rate and the rate of disability progression [1], [2], [3], [4].

The concept of treatment adherence encompasses both compliance and persistence. Compliance can be defined as the ability to follow a pre-specified administration schedule without missing doses, which was not assessed in the current study. Persistence refers to a patient’s ongoing treatment utilisation [5].

Our current understanding of DMT persistence in MS has been informed by data from a number of sources, including large phase III trials [6]. However, the environment of a trial does not reflect typical clinical practice. Many clinical practice-based DMT utilisation studies have been retrospective or relied on insurance claims or prescription data [7], [8], [9]. In general, these studies have shown poor persistence rates for MS DMTs, but they are methodologically weakened by bias due to their retrospective nature and limited clinical data [10], [11].

Therefore, we chose to undertake a large, prospective, multicentre study of MS therapy utilisation (encompassing persistence), and predictors of treatment switching and discontinuation. We sought to assess persistence and switch on all commercially available DMT including IFNβ, GA and NAT all of which have first-line indications in Australia.

## Methods

### MSBase Registry

The MSBase Registry (www.msbase.org) is a collaborative international registry that prospectively collects neurological outcome data from consenting MS patients attending MS specialist centres and clinics [12]. The registry is operated by the not-for-profit MSBase Foundation and its data is physician-owned, with access freely available to participating neurologists.

Data are collected using an offline, electronic medical record program called iMed within clinical settings. Quality control of data is ensured through the use of drop down menus restricting data entry errors. Data are then anonymised and transmitted to the MSBase Registry server. The MSBase Registry contains data collected from over 65 clinics in 28 countries, representing over 20,000 patient datasets. For quality assurance, all participating neurologists are required to complete online Expanded Disability Status Scale (EDSS) certification through the Neurostatus online certification program (www.neurostatus.net).

### The Australian MSBase Clinical Cohort

Patients with MS (revised McDonald criteria) were enrolled from seven Australian academic centres with specialist MS clinics (The Royal Melbourne Hospital, Vic; Box Hill Hospital, Vic; John Hunter Hospital, NSW; Brain and Mind Research Institute, NSW; Flinders Medical Centre, SA; Geelong Hospital, Vic, and Westmead Hospital, NSW). Patients underwent routine clinical assessments, were subtyped by clinical course and had the MSBase minimum dataset updated during routine initial and follow-up clinic visits [12]. Follow-up visits occurred at least once annually. Centres provided patients with access to multidisciplinary care, including nurse education for GA and IFN injection training and regular follow-up by nurses post initial training.

### Data Collection

Treating physicians prospectively collected pre-specified data at the time of clinic visit.

Treatment start, stop and switch decisions were made in consultation between patients and their treating physicians during clinic visits and all treatment identities, start and stop dates were recorded at that time.

When patients discontinued treatment, a field with categorical reasons for treatment discontinuation appeared upon entering a treatment stop date. The reporting of reasons for treatment discontinuation was not mandated for this analysis, however, all collected reasons for treatment discontinuation were analysed (52% of all discontinuations). The captured discontinuation reasons were balanced across all DMT preparations.

The observation period for this study commenced on 1 January 1998, when MS-specific DMTs became commercially available in Australia, and ended on the date of data extraction, 10 June 2010. As MSBase is an ongoing project, data were censored at the patients’ most recent visit. A minimum of 2 visits per patient were required to be included in the study; therefore, the observation interval for each patient was defined by their first and last visits. Patients with only a single visit recorded were excluded from analysis. Patients who initiated with their first DMT prior to 1998 were excluded from this study. Only new users were included in this analysis.

Data extracted from the MSBase Registry on 10 June 2010 comprised 1618 Australian patient datasets, representing approximately 15% of the Australian MS patient population. These patients were typical of those seen in large tertiary referral centres.

### Ethics Statement

All patients gave written informed consent to participate in the MSBase Registry and Human Research Ethics Committee (HREC) approval was obtained from all participating centres: The Royal Melbourne Hospital; Box Hill Hospital; John Hunter Hospital; Brain and Mind Research Institute; The Southern Adelaide Clinical HREC; Barwon Health; Western Sydney Local Health District.

### Statistical Analyses

Sex, age, disease course, DMT identity, reasons for treatment discontinuation, proportion of time treated (PTT) and annualised switch rates were summarised using frequencies and percentages. As EDSS, treatment persistence and time to treatment switch all demonstrated non-normality, these were described using medians and inter-quartile ranges (IQR). Data assessing treatment duration were censored at the patients’ most recent clinic visit date. Kaplan-Meier estimates were used to describe the cumulative probability of treatment discontinuation. Predictors of treatment discontinuation were analysed using univariable and multivariable Cox proportional hazards regression and quantified using Hazard Ratios (HR). Hazard proportionality was assessed by analysis of scaled Schoenfeld residuals. One-way ANOVA with Bonferroni’s post hoc test was used to test for differences between continuous variables, χ^2^ tests were used for categorical variables and Kruskal-Wallis tests were used to test for differences between discrete variables.

All reported p values are two-tailed and for each analysis p<0.05 was considered significant. All analyses were performed using Stata version 12.0 software package (StataCorp, College Station, Texas).

### Definitions

PTT was the proportion of time patients were treated with a DMT as recorded in the MSBase Registry. The PTT assumes compliance.

Treatment cessation was defined as a break of 90 or more days with no further DMT use recorded. Patients were considered to have switched if a subsequent DMT was recorded.

## Results

### Australian MS Clinical Cohort Demographics

The Australian MSBase Registry cohort had a median follow up of 2.3 years (IQR: 1.0, 4.4 years). At the date of data extraction, the average age of the cohort was 45.4 (standard deviation, SD) 12.5 years, comprising 74.4% females and 25.6% males with a median EDSS of 3 (range 0–9).

The cohort consisted of patients with clinically isolated syndrome (CIS) 6.0%, RRMS 68.8%, secondary progressive MS (SPMS) 15.8%, primary progressive MS (PPMS) 6.6% and progressive relapsing MS (PRMS) 2.8%. For the purposes of the current study, only RRMS patients were further analysed.

### RRMS Patient Demographics

A total of 1113 RRMS patients were followed up for a median of 2.0 years (IQR 0.80, 4.0). The RRMS population comprised 856 (76.9%) females and 257 (23.1%) males who were predominantly of Caucasian background (95.3%), with the remaining 4.7% comprising Semitic, Asian, Eurasian, Hispanic, African and Inuit backgrounds. The average age at RRMS diagnosis was 31.8 (SD 10.1) years, with median time to diagnosis of 1.1 years (IQR: 0.33, 3.9 years).

### Overview of Treatment Utilisation by RRMS Patients

Median time to treatment initiation with DMTs for patients diagnosed with RRMS was 0.64 years (IQR 0.16, 3.16) from clinically definite MS diagnosis date. A total of 724 (65%) RRMS patients were treated with DMTs at their most recent visit. A total of 908 (81.6%) patients had used at least one DMT at some point, while 205 patients (18.4% of the RRMS population) were never treated with a DMT.

Of the patients treated with a DMT at their most recent visit, 80.4% were treated with either GA or an IFNβ preparation, 18.5% were treated with NAT, and 1.8% were treated with chemotherapeutics.

We recorded 771 first GA/IFNβ/NAT treatment commencements. These patients were followed up for a median of 4.2 years (IQR 2.1, 7.0), and met with their treating physician a median of 6 times (IQR 3, 11) over the observation period. Baseline characteristics of patients initiating with their first DMT are summarised in Table 1. Of these 771 first treatment initiations, only 325 (42.2%) patients were still continuing on their first DMT at their most recent visit, while 105 (13.6%) patients ceased all treatments and 43 patients (5.6%) recorded a delayed continuation (defined as a break from their DMT of 90 days or more only to re-initiate the same agent). Switch of therapy was recorded in 298 (38.6%) patients.

**Table 1:** Baseline Patient Characteristics at first treatment initiation.

	All treatments n = 771	IFNβ-1a IM n = 153	IFNβ-1a SC n = 220	IFNβ-1b n = 270	GA n = 117	NAT n = 11	p-value Between treatment groups
**Female n (%)**	592 (76.8)	110 (71.9)	168 (76.4)	216 (80.0)	92 (78.6)	6 (54.6)	0.140α
**Age at MS onset, y mean (SD)**	31.4 (10.0)	31.8 (10.4)	31.0 (9.8)	30.9 (9.9)	33.1 (9.9)	27.8 (9.4)	0.192β
**Age at treatment start, y mean (SD)**	36.8 (10.7)	37.6 (11.0)	35.9 (10.6)β	36.0 (10.7)β	39.6 (10.0)β	35.3 (12.4)	0.024β
**Disease duration at treatment start, y median (IQR)**	2.7 (0.9, 7.7)	2.5 (0.9, 8.4)	2.5 (0.9, 7.0)	2.5 (0.8, 7.1)	3.3 (1.1, 9.2)	6.2 (3.2, 11.5)	0.117γ
**EDSS at treatment start median (IQR)**	2 (1, 3)	2 (0, 2.5)	2 (1, 3)	2 (1, 3.5)	2 (1, 3)	3 (1.5, 5.5)	0.154γ

Abbreviations: n, number; y, years; SD, standard deviation; IQR, interquartile range; EDSS, Expanded Disability Status Scale.

α Pearson χ^2^ test.

β One-way ANOVA with Bonferroni’s post hoc test.

γ Kruskal-Wallis rank sum test.

The median duration off therapy when switching from a first GA/IFNβ therapeutic to a second GA/IFNβ/NAT DMT was 28 days (IQR 1, 122), whereas median time off therapy for a delayed continuation was 452 days (IQR 273, 802).

The female/male ratio for the entire RRMS cohort was 3.3∶1. For patients continuing on treatment, the sex ratio was 3.1∶1; for patients switching treatment it was 3.1∶1; for patients disengaging from treatment the sex ratio was 3.4∶1 and for patients who recorded a delayed continuation, the sex ratio was 13.3∶1 (Pearson χ^2^, p = 0.08), as the vast majority of these were due to pregnancy.

### DMT Persistence

#### First treatment initiation

**Figure 1:**
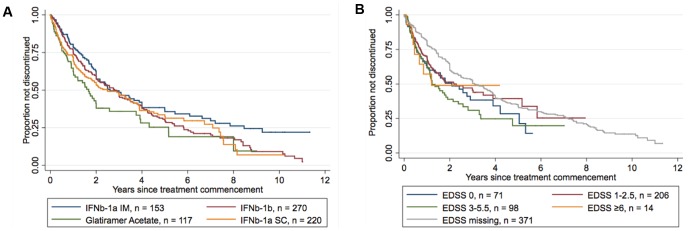
Kaplan-Meier survival estimates for treatment discontinuation (First DMT). A: Treatment discontinuation by DMT. This figure demonstrates that patients prescribed Glatiramer Acetate as a first DMT discontinue treatment at a significantly greater rate than those prescribed any of the IFNβ preparations (adjusted Cox Proportional Hazards Regression, p<0.03). B: Treatment discontinuation by EDSS. This figure demonstrates that patients with an EDSS of 3–5.5 discontinue the use of a first DMT at a greater rate than those with an EDSS of 0 (adjusted Cox Proportional Hazards Regression, p = 0.08).

Australian RRMS patients persisted on their first GA/IFNβ/NAT DMT for a median duration of 2.5 years (IQR 1.0, 6.7, n = 771). When analysed by individual DMT, patients remained on GA for a median of 1.7 years (IQR 0.62, 5.2, n = 117), IFNβ-1a IM for a median of 2.6 years (IQR 1.2, 8.5, n = 153), IFNβ-1b for a median of 2.8 years (IQR 1.0, 5.8, n = 270), and IFNβ-1a SC for a median of 2.5 years (IQR 0.70, 7.1, n = 220; Figure 1a). NAT was a rare first choice of DMT. These patients were followed-up on therapy for an average of 1.2 years (SD 0.72 years, n = 11). No patients had ceased first-line NAT treatment at their most recent visit.

Table 2 summarises both univariable and multivariable analyses of predictors of treatment discontinuation of first recorded DMT. We found on both unadjusted and adjusted analyses that patients receiving GA as their first DMT discontinued treatment at a greater rate than those patients on IFNβ-1a IM (HR 1.74, p = 0.001 on adjusted analysis). Similarly, patients initiating with GA as their first DMT discontinued treatment at a greater rate than patients on IFNβ-1b (HR 1.47, p = 0.01) or IFNβ-1a SC (HR 1.40, p = 0.03, Table S1).

**Table 2:** Predictors of first treatment discontinuation.

Predictor	Level	Discontinuations n = 460	Unadjustedα HR (95% CI)p-value	Adjusted^α#^ HR (95% CI) p-value
**Demographics**				
*Sex*	Female	359	1.00	1.00
	Male	101	1.02 (0.82, 1.27) 0.883	0.99 (0.79, 1.24) 0.935
*Disease duration at treatment start*	per 10 years	−	**0.84 (0.72, 0.98) 0.029**	0.96 (0.81, 1.14) 0.670
*Age at treatment start*	per 10 years	−	**0.81 (0.74, 0.89) 0.000**	**0.79 (0.71, 0.87) 0.000**
**Treatment**				
	IFNb-1a IM	83	1.00	1.00
	IFNb-1b	179	1.28 (0.99, 1.67) 0.061	1.19 (0.91, 1.55) 0.199
	IFNb-1a SC	133	**1.33 (1.01, 1.76) 0.042**	1.24 (0.94, 1.65) 0.130
	GA	65	**1.75 (1.26, 2.43) 0.001**	**1.74 (1.25, 2.42) 0.001**
**EDSS**				
*EDSS (categorical) at treatment start*	0	40	1.00	1.00
	1–2.5	91	0.89 (0.61, 1.28) 0.522	0.98 (0.67, 1.43) 0.913
	3–5.5	57	1.19 (0.80, 1.79) 0.390	1.45 (0.96, 2.20) 0.078
	≥6	7	0.92 (0.41, 2.04) 0.829	1.16 (0.51, 2.66) 0.717
	missing*	265	**0.70 (0.50, 0.98) 0.036**	0.78 (0.56, 1.10) 0.163

Abbreviations: n: number, HR: Hazard Ratio, CI: Confidence Interval, IFN: Interferon, IM: intramuscular, SC: Subcutaneous, GA: Glatiramer Acetate, EDSS: Expanded Disability Status Scale.

Treatment initiations n = 760 excluding Natalizumab (n = 11).

α Cox Proportional Hazards Regression.

Multivariable Cox Proportional Hazards model was adjusted for sex, disease duration, age at treatment start, treatment and EDSS.

# Proportional hazards test: p = 0.3747.

* No EDSS score available at the time of treatment start.

Adjusted, multivariable modelling further revealed that older age at treatment start (HR 0.79 per 10 years, p<0.000) was an independent predictor of treatment persistence, and an EDSS of 3.0–5.5 as compared to an EDSS of zero approached significance as an independent predictor of treatment discontinuation (Figure 1b). Disease duration at the time of treatment initiation was shown to predict treatment persistence using univariable analysis, but it was no longer significant in the adjusted multivariable analysis. Sex was not predictive of treatment persistence in this RRMS population (Table 2).

#### Second and subsequent treatment initiation

**Figure 2:**
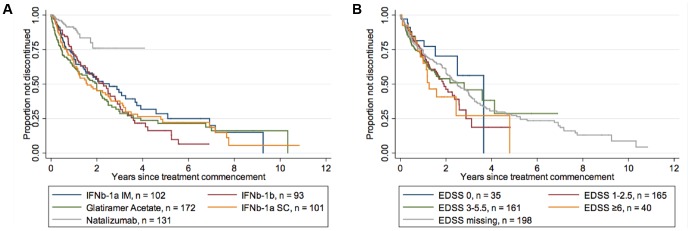
Kaplan-Meier survival estimates for treatment discontinuation (Subsequent DMT). A: Treatment discontinuation by DMT. This figure demonstrates that patients prescribed Natalizumab as a subsequent DMT discontinue treatment at a significantly slower rate than those prescribed Glatiramer Acetate or any of the IFNβ preparations (adjusted Cox Proportional Hazards Regression, p = 0.000). B: Treatment discontinuation by EDSS. This figure demonstrates that patients with EDSS 1–2.5 (p = 0.046), EDSS 3–5.5 (p = 0.013) and EDSS ≥6 (p = 0.008) discontinue treatment at a significantly greater rate than those with EDSS 0 (adjusted Cox Proportional Hazards Regression).

Baseline patient characteristics at initiation of second or subsequent DMT are summarised in Table 3. Patients persisted on a second or subsequent GA/IFNβ/NAT DMT for a median of 2.3 years (IQR 0.87, 4.9, n = 599). When analysed by individual DMT, patients remained on GA for a median of 2.0 years (IQR 0.45, 3.9, n = 172), IFNβ-1a IM for a median of 2.5 years (IQR 0.7, 5.1, n = 102), IFNβ-1b for a median of 2.3 years (IQR 0.90, 3.6, n = 93), and IFNβ-1a SC for a median of 1.6 years (IQR 0.67, 4.8, n = 101). Patients were followed-up on NAT for an average of 1.2 years (SD 0.67 years, n = 131). NAT discontinuation events were rare (n = 18). Adjusted Cox proportional hazards regression revealed that the hazard ratio for treatment discontinuation was 0.26 (p<0.000; Table 4 and Figure 2a) if using NAT as compared to IFNβ-1a IM. Similarly, the hazard ratio for treatment discontinuation was significantly smaller (p<0.000) for NAT as compared to IFNβ-1b, IFNβ-1a SC and GA (see Table S2). There were no differences in treatment persistence between IFNβ preparations or GA when used as a second or subsequent DMT.

**Table 3:** Baseline Patient Characteristics at subsequent treatment initiation.

	All treatments n = 599	IFNβ-1a IM n = 102	IFNβ-1a SC n = 101	IFNβ-1b n = 93	GA n = 172	NAT n = 131	p-value Between treatment groups
**Female n (%)**	482 (80.5)	83 (81.4)	83 (82.2)	83 (89.3)	140 (81.4)	93 (71.0)	0.015α
**Age at MS onset, y mean (SD)**	28.6 (9.3)	28.2 (8.3)	27.4 (9.8)	28.5 (8.8)	30.1 (10.3)	28.0 (8.2)	0.125β
**Age at treatment start, y mean (SD)**	37.5 (10.1)	37.3 (9.5)	36.9 (10.7)	36.1 (9.7)	38.6 (10.7)	37.7 (9.4)	0.386β
**Disease duration at treatment start, y median (IQR)**	7.2 (3.4, 12.4)	7.0 (3.2, 13.7)	7.7 (3.6, 12.4)	6.4 (2.8, 10.4)	6.5 (3.1, 11.7)	8.1 (4.4, 12.9)	0.137γ
**EDSS at treatment start median (IQR)**	3 (2,4)	2.5 (1.5, 3.75)γ	2.5 (1.5, 4)γ	2.5 (1.5, 4)γ	2.5 (2, 4)γ	4 (2.5, 4.5)γ	0.0001γ

Abbreviations: n, number; y, years; SD, standard deviation; IQR, interquartile range; EDSS, Expanded Disability Status Scale.

α Pearson χ^2^ test.

β One-way ANOVA with Bonferroni’s post hoc test.

γ Kruskal-Wallis rank sum test.

**Table 4:** Predictors of subsequent treatment discontinuation.

Predictor	Level	Discontinuations n = 296	Unadjustedα HR (95% CI) p-value	Adjusted^α#^ HR (95% CI) p-value
**Demographics**				
*Sex*	Female	241	1.00	1.00
	Male	55	1.02 (0.76, 1.36) 0.920	1.11 (0.82, 1.49) 0.512
*Disease duration at treatment start*	per 10 years	−	1.03 (0.87, 1.23) 0.742	1.10 (0.90, 1.33) 0.345
*Age at treatment start*	per 10 years	−	0.93 (0.83, 1.04) 0.205	**0.85 (0.75, 0.97) 0.017**
**DMT**				
**Therapeutic**	IFNb-1a IM	56	1.00	1.00
	IFNb-1b	59	1.18 (0.82, 1.71) 0.356	1.09 (0.75, 1.58) 0.657
	IFNb-1a SC	66	1.18 (0.83, 1.69) 0.354	1.10 (0.77, 1.57) 0.611
	GA	97	1.29 (0.93, 1.79) 0.133	1.25 (0.90, 1.74) 0.190
	NAT	18	**0.37 (0.21, 0.63) 0.000**	**0.26 (0.15, 0.45) 0.000**
**EDSS**				
*EDSS (categorical) at treatment start*	0	10	1.00	1.00
	1–2.5	67	1.65 (0.85, 3.21) 0.139	**1.98 (1.01, 3.86) 0.046**
	3–5.5	61	1.54 (0.79, 3.02) 0.203	**2.40 (1.20, 4.81) 0.013**
	≥6	18	1.98 (0.91, 4.28) 0.084	**2.90 (1.31, 6.42) 0.008**
	missing*	140	1.32 (0.69, 2.52) 0.403	1.34 (0.70, 2.58) 0.379

Abbreviations: n: number, HR: Hazard Ratio, CI: Confidence Interval, IFN: Interferon, IM: intramuscular, SC: Subcutaneous, GA: Glatiramer Acetate, NAT: Natalizumab, EDSS: Expanded disability status scale.

Treatment initiations n = 599.

α Cox Proportional Hazards Regression.

Multivariable Cox Proportional Hazards model was adjusted for sex, disease duration, age at treatment start, treatment and EDSS.

# Proportional hazards test: p = 0.2270.

* No EDSS score available at treatment start.

Adjusted multivariable analyses revealed that older age at treatment start was again predictive of treatment persistence (HR 0.85 per 10 years, p = 0.017). Additionally, EDSS at treatment start was independently predictive of treatment discontinuation on a subsequent DMT. EDSS 1–2.5 (HR 1.98, p = 0.046), EDSS 3–5.5 (HR 2.40, p = 0.013), and EDSS 6+ (HR 2.90, p = 0.008) were associated with greater rates of discontinuation relative to an EDSS of 0 (Table 4 and Figure 2b). Disease duration at treatment start and sex were not predictive of treatment discontinuation on a second or subsequent DMT (Table 4).

### Treatment Discontinuation

While the recording of reasons for treatment discontinuation does not constitute part of the MSBase minimum dataset, categorical reasons were collected for approximately 52% of all discontinuations (see Table 5). Reasons included: lack of tolerance/adverse event, convenience, lack of improvement, progression of disease and scheduled stop. Here we report that by far the most common reason for treatment discontinuation was lack of tolerance/adverse event (42.9%–64.7% of all responses). There were no statistically significant differences between DMTs for any categorical discontinuation descriptors (Table 5).

**Table 5:** Categorical reasons for treatment discontinuation for all treatment commencements.

	IFNβ-1a IM	IFNβ-1b	IFNβ-1a SC	GA	NAT	p-value Between treatment groups
**No. of Commencements – n**	465	702	499	529	243	
**No. of Discontinuations – n**	276	474	307	286	56	
**No. of Recorded Reasons for Discontinuation – n (%)**	128 (46.4)	228 (48.1)	150 (48.9)	155 (54.2)	35 (62.5)	
**Reasons Recorded – n (%)**						
*Adverse Event/Lack of Tolerance*	66 (51.6)	125 (54.8)	97 (64.7)	95 (61.3)	15 (42.9)	0.345α
*Convenience*	5 (3.9)	17 (7.5)	6 (4.0)	9 (5.8)	6 (17.1)	0.035α
*Lack of Improvement*	16 (12.5)	24 (10.5)	18 (12.0)	20 (12.9)	4 (11.4)	0.447α
*Progression of Disease*	23 (18.0)	34 (14.9)	16 (10.7)	22 (14.2)	7 (20.0)	0.382α
*Scheduled Stop*	18 (14.0)	28 (12.3)	13 (8.6)	9 (5.8)	3 (8.6)	0.061α
**Total**	128 (100)	228 (100)	150 (100)	155 (100)	35 (100)	

Abbreviations: n, number.

α Pearson χ^2^ test.

### Proportion of Time Treated, Treatment Cessations and Switches

Annualised PTT was calculated for all DMTs based on treatment commencements and cessations between 11 June 2008 to 10 June 2010, the date of data extract. There were 535 patients who were treated with DMT during this observation period, with a median annualised PTT of 0.87 (IQR 0.47, 1).

Annualised treatment switch rates were calculated based on treatment commencements and cessations for each individual drug over the same two-year period as above. The annualised treatment switch rates for these DMTs were as follows: IFN-1a IM, 9.5% per annum; IFN-1b, 12.5% per annum; GA, 11.6% per annum; IFN-1a SC, 10.0% per annum and NAT 4.4% per annum.

Concerning switches within the interferon class, there was no evidence to suggest preferential switching (Table 6). Patients switching from IFNβ preparations switched to another interferon class (44.4%), to GA (36.6%) or to NAT (19.0%). The majority of patients switching from GA changed to an IFNβ preparation (73.7%) while just over a quarter of patients treated with GA initially changed to NAT (26.3%). At the time of data extraction, no patients for whom NAT was their first-recorded DMT (n = 11) had switched treatment.

**Table 6:** Proportion of patients class switching from first to second IFNβ preparation.

		First IFNβ		
Second IFNβ		IFNβ-1a IM	IFNβ-1b	IFNβ-1a SC
	IFNβ-1a IM	−	63.9%	51.7%
	IFNβ-1b	45.5%	−	48.3%
	IFNβ-1a SC	54.5%	36.1%	−
	Total	100%	100%	100%

## Discussion

Poor adherence to long term therapies in a chronic disease such as MS is thought to be a major contributor to the health care burden and, conversely, improved adherence to extant therapies is postulated to potentially have a greater impact on health outcomes than the development of new therapeutics [13].

Use of disease modifying therapies in MS has been shown in randomised controlled trials (RCT) to decrease relapse rate and disability progression [3], [4], [14], [15]. In a global survey commissioned by the World Health Organisation, it was reported that the median percentage of patients in high income countries receiving DMT is 75% [16]. Concordant with this report, in our study we show that 65% of eligible patients in our registry were treated at the time of data extraction and that in the most recent two-year observation period, our patients prescribed DMT spent 87% of this time on treatment. However, we also found that 18.4% of RRMS patients had never engaged with treatment. The proportion of RRMS patients not engaging with treatment likely represents a heterogeneous group comprising some with relatively mild disease, and others choosing not to engage with parentally administered treatments. With the recent approval by the Therapeutics Goods Administration of the oral therapeutic fingolimod in Australia from September 2011, and with other oral therapeutics in the pipeline, it will be interesting to determine whether the proportion of RRMS patients engaging with DMT will increase in the future.

This is the first large-scale, multicentre, prospective study of MS treatment utilisation in Australia. The Australian health care system, through the Pharmaceutical Benefits Scheme, provides drug cost coverage for RRMS patients for all IFNβ preparations, GA and NAT, removing confounding issues of drug cost and insurance coverage from the current analysis. Most previous studies of treatment persistence in MS have used retrospective administrative claims data [7], [8], [9], [17]. In comparison, a key strength of our study is its prospective nature. Outcomes data are recorded at the time of the clinical encounter into an electronic medical record by MS clinicians, eliminating recall bias, transcriptional errors and avoiding duplicate records. However, this study comprises datasets of patients seen at academic centres who have volunteered to participate in the MSBase Registry, therefore this study is subject to selection bias.

In Australia, GA, IFNβ and NAT all have first-line indications with no differences in criteria for use, therefore switch decisions were at the discretion of the treating physician and their patient. In the current study, treatment persistence on immunotherapy was relatively short and therapy switches were common. We report a median duration of only 2.6 years on a first DMT and 2.3 years on a subsequent DMT. Interestingly, product identity was associated with different treatment discontinuation rates where patients receiving NAT as a subsequent DMT were more likely to persist on treatment as compared to those on IFNβ or GA; and patients receiving GA as their first DMT were more likely to cease treatment than patients on IFNβ-1a IM. These prospective data are concordant with that reported by Kleinman et al [8] who found, using retrospective US healthcare claims data, that IFNβ preparations were associated with a lower discontinuation rate than GA. However, this finding is to some extent in contrast to the adherence outcome of our previous global study [18] and the REGARD study [19]. In the REGARD study, the overall persistence rate for GA and IFNβ-1a SC over 96 weeks was similar, at around 80%. This discrepancy also highlights a major difference in persistence rates reported in clinical trials, such as REGARD, compared to reports from country-specific clinical practice studies, such as the present study or the recently published Ontario data [20], which report persistence rates of less than 50% at three years and two years post DMT initiation.

Treatment adherence and persistence rates are known to be influenced by country of residence [9], [21], [22] with higher adherence rates reported in Italy and Spain compared to Canada and Australia. Common comorbidities such as depression also influence persistence [23]. In a global analysis of DMT utilisation in CIS and early RRMS in the MSBase registry [18], females were more likely to discontinue than males. This was not replicated in our Australian cohort, with males and females being equally likely to cease first DMT. Consistent with previous registry studies, we report that patients with a higher EDSS were more likely to discontinue treatment [18], [21], [22]. Additionally, we found that older patients were more likely to persist on therapy than younger patients independent of treatment identity or order.

The reasons for DMT discontinuation are rarely identified in the persistence literature, but three studies report poor tolerability, perceived lack of efficacy and adverse events as reasons for discontinuation [9], [21], [22]. Concordant with these results, by far the most common reason for DMT discontinuation in our study was lack of treatment tolerance or treatment-related adverse event.

A limitation of the current study is the incomplete capture of causes for treatment discontinuations (52% of all discontinuations were assigned a reason). However, the data available are evenly distributed across all the therapies and therefore likely to be representative of causes for treatment cessation. Additionally, whilst compliance was encouraged in our centres through the provision of immunotherapy training and regular nurse follow-up post-injection training, we were not able to ascertain compliance in this study.

In summary, in this prospective multicentre study, we have demonstrated that in Australian tertiary centres with specialist MS care clinics, median treatment persistence is relatively short; 2.5 years first initiation, 2.3 years subsequent initiation. We report that older age at treatment initiation is associated with greater treatment persistence and that higher EDSS is independently predictive of treatment discontinuation. We further report that treatment persistence was poorest on GA as a first DMT, however, there were no differences in treatment persistence between GA or any of the IFNβ preparations as subsequent therapies. Treatment persistence was greatest for NAT. The most common reason for treatment discontinuation is poor treatment tolerability. Overall, this clinical practice-based study highlights the significant unmet need to develop effective MS therapies with improved tolerability, and to implement country-specific strategies that enhance medication persistence in this chronic, life-long condition.

## Supporting Information

Table S1 **Predictors of first treatment discontinuation.** Table reports univariable and multivariable Cox proportional hazards regression analysis. Comparator group: GA-treated patients.
(DOCX)

Table S2 **Predictors of subsequent treatment discontinuation.** Table reports univariable and multivariable Cox proportional hazards regression analysis. Comparator group: NAT-treated patients.
(DOCX)
