# Supplementary material for: The Australian Multiple Sclerosis (MS) Immunotherapy Study: A Prospective, Multicentre Study of Drug Utilisation Using the MSBase Platform
Source: PLoS One. 2013 Mar 19;8(3):e59694. doi: 10.1371/journal.pone.0059694 (PMC3602083; doi:10.1371/journal.pone.0059694)
Supplement: Table S1 — Predictors of first treatment discontinuation. Table reports univariable and multivariable Cox proportional hazards regression analysis. Comparator group: GA-treated patients. (DOCX) [file pone.0059694.s001.docx]

**Table S1. Predictors of first treatment discontinuation. Comparator group: GA-treated patients.**

| **Predictor** | **Level** | **Discontinuations**  **n = 460** | **Unadjusted^α^**  **HR (95% CI) p-value** | **Adjusted^α#^**  **HR (95% CI) p-value** |
| --- | --- | --- | --- | --- |
| **Demographics** |  |  |  |  |
| *Sex* | Female | 359 | 1.00 | 1.00 |
|  | Male | 101 | 1.02 (0.82, 1.27) 0.883 | 0.99 (0.79, 1.24) 0.935 |
| *Disease duration at treatment start* | per 10 years | - | **0.84 (0.72, 0.98) 0.029** | 0.96 (0.81, 1.14) 0.670 |
| *Age at treatment start* | per 10 years | - | **0.81 (0.74, 0.89) 0.000** | **0.79 (0.71, 0.87) 0.000** |
| **Treatment** |  |  |  |  |
|  | IFNb-1a IM | 83 | **0.57 (0.41, 0.79) 0.001** | **0.57 (0.41, 0.80) 0.001** |
|  | IFNb-1b | 179 | **0.73 (0.55, 0.98) 0.033** | **0.68 (0.51, 0.91) 0.010** |
|  | IFNb-1a SC | 133 | 0.76 (0.57, 1.03) 0.073 | **0.71 (0.53, 0.96) 0.028** |
|  | GA | 65 | 1.00 | 1.00 |
| **EDSS** |  |  |  |  |
| *EDSS (categorical) at treatment start* | 0 | 40 | 1.00 | 1.00 |
|  | 1-2.5 | 91 | 0.89 (0.61, 1.28) 0.522 | 0.98 (0.67, 1.43) 0.913 |
|  | 3-5.5 | 57 | 1.19 (0.80, 1.79) 0.390 | 1.45 (0.96, 2.20) 0.078 |
|  | 6+ | 7 | 0.92 (0.41, 2.04) 0.829 | 1.16 (0.51, 2.66) 0.717 |
|  | missing | 265 | **0.70 (0.50, 0.98) 0.036** | 0.78 (0.56, 1.10) 0.163 |

Abbreviations: n: number, HR: Hazard Ratio, CI: Confidence Interval, IFN: Interferon, IM: intramuscular, SC: Subcutaneous, GA: Glatiramer Acetate, EDSS: Expanded Disability Status Scale

Treatment initiations n = 760 excluding Natalizumab (n=11)

^α^Cox Proportional Hazards Regression

Multivariable Cox Proportional Hazards model was adjusted for sex, disease duration, age, treatment and EDSS

# Proportional hazards test: p=0.3747
