# Supplementary material for: The Australian Multiple Sclerosis (MS) Immunotherapy Study: A Prospective, Multicentre Study of Drug Utilisation Using the MSBase Platform
Source: PLoS One. 2013 Mar 19;8(3):e59694. doi: 10.1371/journal.pone.0059694 (PMC3602083; doi:10.1371/journal.pone.0059694)
Supplement: Table S2 — Predictors of subsequent treatment discontinuation. Table reports univariable and multivariable Cox proportional hazards regression analysis. Comparator group: NAT-treated patients. (DOCX) [file pone.0059694.s002.docx]

**Table S2. Predictors of subsequent treatment discontinuation. Comparator group: NAT-treated patients.**

| **Predictor** | **Level** | **Discontinuations**  **n = 296** | **Unadjusted^α^**  **HR (95% CI) p-value** | **Adjusted^α#^**  **HR (95% CI) p-value** |
| --- | --- | --- | --- | --- |
| **Demographics** |  |  |  |  |
| *Sex* | Female | 241 | 1.00 | 1.00 |
|  | Male | 55 | 1.02 (0.76, 1.36) 0.920 | 1.11 (0.82, 1.49) 0.512 |
| *Disease duration at treatment start* | per 10 years | - | 1.03 (0.87, 1.23) 0.742 | 1.10 (0.90, 1.33) 0.345 |
| *Age at treatment start* | per 10 years | - | 0.93 (0.83, 1.04) 0.205 | **0.85 (0.75, 0.97) 0.017** |
| **DMT** |  |  |  |  |
| **Therapeutic** | IFNb-1a IM | 56 | **2.72 (1.59, 4.66) 0.000** | **3.85 (2.20, 6.72) 0.000** |
|  | IFNb-1b | 59 | **3.24 (1.90, 5.51) 0.000** | **4.19 (2.42, 7.23) 0.000** |
|  | IFNb-1a SC | 66 | **3.22 (1.90, 5.47) 0.000** | **4.22 (2.45, 7.28) 0.000** |
|  | GA | 97 | **3.50 (2.11, 5.82) 0.000** | **4.81 (2.84, 8.12) 0.000** |
|  | NAT | 18 | 1.00 | 1.00 |
| **EDSS** |  |  |  |  |
| *EDSS (categorical) at treatment start* | 0 | 10 | 1.00 | 1.00 |
|  | 1-2.5 | 67 | 1.65 (0.85, 3.21) 0.139 | **1.98 (1.01, 3.86) 0.046** |
|  | 3-5.5 | 61 | 1.54 (0.79, 3.02) 0.203 | **2.40 (1.20, 4.81) 0.013** |
|  | 6+ | 18 | 1.98 (0.91, 4.28) 0.084 | **2.90 (1.31, 6.42) 0.008** |
|  | missing | 140 | 1.32 (0.69, 2.52) 0.403 | 1.34 (0.70, 2.58) 0.379 |

Abbreviations: n: number, HR: Hazard Ratio, CI: Confidence Interval, IFN: Interferon, IM: intramuscular, SC: Subcutaneous, GA: Glatiramer Acetate, NAT: Natalizumab, EDSS: Expanded disability status scale

Treatment initiations n = 599

^α^Cox Proportional Hazards Regression

Multivariable Cox Proportional Hazards model was adjusted for sex, disease duration, age, treatment and EDSS

# Proportional hazards test: p=0.2270
